# Pluripotent Stem Cells as a Model for Human Embryogenesis

**DOI:** 10.3390/cells12081192

**Published:** 2023-04-20

**Authors:** Daniela Ávila-González, Mikel Ángel Gidi-Grenat, Guadalupe García-López, Alejandro Martínez-Juárez, Anayansi Molina-Hernández, Wendy Portillo, Néstor Emmanuel Díaz-Martínez, Néstor Fabián Díaz

**Affiliations:** 1 Laboratorio de Reprogramación Celular y Bioingeniería de Tejidos, Biotecnología Médica y Farmacéutica, Centro de Investigación y Asistencia en Tecnología y Diseño del Estado de Jalisco, Guadalajara 44270, Mexico; 2 Departamento de Fisiología y Desarrollo Celular, Instituto Nacional de Perinatología, Ciudad de México 11000, Mexico; 3 Instituto de Neurobiología, Universidad Nacional Autónoma de México, Querétaro 76230, Mexico

**Keywords:** blastocyst, amnion, cell culture

## Abstract

Pluripotent stem cells (PSCs; embryonic stem cells and induced pluripotent stem cells) can recapitulate critical aspects of the early stages of embryonic development; therefore, they became a powerful tool for the in vitro study of molecular mechanisms that underlie blastocyst formation, implantation, the spectrum of pluripotency and the beginning of gastrulation, among other processes. Traditionally, PSCs were studied in 2D cultures or monolayers, without considering the spatial organization of a developing embryo. However, recent research demonstrated that PSCs can form 3D structures that simulate the blastocyst and gastrula stages and other events, such as amniotic cavity formation or somitogenesis. This breakthrough provides an unparalleled opportunity to study human embryogenesis by examining the interactions, cytoarchitecture and spatial organization among multiple cell lineages, which have long remained a mystery due to the limitations of studying in utero human embryos. In this review, we will provide an overview of how experimental embryology currently utilizes models such as blastoids, gastruloids and other 3D aggregates derived from PSCs to advance our understanding of the intricate processes involved in human embryo development.

## 1. Introduction

Developmental biology has the challenge of deciphering the molecular mechanisms that control cellular processes during embryogenesis and morphogenesis and their interactions to determine precise time–space patterns and how a living organism is generated. The first milestones in the field were experiments on amphibian embryos (genus *Triturus*) performed by Hans Spemann and his disciple Hilda Mangold, more than a century ago. They described a cellular region capable of instructing the formation of specific lineages and an embryonic axis in the early gastrula stage. Thus, the discovery of the now-called Spemann–Mangold organizer was one of the pillars of experimental and comparative embryology to define essential concepts of developmental biology, such as induction, specification, competition, and differentiation. A new wave of experimental approaches, in which different vertebrate animals are genetically modified (transgenic models), either for cell lineage tracking through genetic labeling or to analyze the effect of gene loss during development (knockout), grew in the last three decades. One of the latest power tools for contrasting data from in vivo and in vitro systems is the use of pluripotent stem cells (PSCs), which are undifferentiated populations that can self-renew and differentiate into every cell type in an organism. Thus, it is possible to elucidate the critical molecular mechanisms that underlie developmental biological processes and extrapolate them to understand vertebrate embryogenesis.

Despite similarities between primate and murine embryonic development, there are notable differences in timing and space. For example, primate pluripotency is more prolonged than the murine model [1]. The human epiblast can also develop into non-embryonic lineages (trophectoderm or hypoblast), but this competence is more restricted in mice [2,3]. Moreover, although the extraembryonic mesoderm and amniotic epithelium lineages develop during implantation in primates, this process occurs only until gastrulation in murine species [4]. On the other hand, Macaca and Callithrix embryos are commonly used as primate models for studying differentiation and development, including humans. However, the limited availability of primate embryos, technical challenges, ethical concerns and the inaccessibility of studying the peri- and post-implantation stages contributed to a lack of understanding of human embryonic development.

Exceptionally, researchers simulated implantation and gastrulation using a defined platform in vitro, thus reaching a significant milestone in the study of ex vivo human embryos [5,6,7]. However, the routine use of embryos in the laboratory is not feasible because of both local and international laws and public judgments regarding the moral status of embryos. As mentioned, human PSCs (hPSCs) are a valuable model for discovering the rules that command what embryonic cells can do and how they do it in a specific time window during development. Indeed, three-dimensional (3D) structures derived from hPSCs can mimic either the blastocyst or particular processes, such as amniogenesis or symmetry breaking, providing new and valuable information about early human embryogenesis.

Here, we describe the period of human development between the blastocyst stage and the beginning of morphogenesis, focusing on pluripotency, hPSC line derivation, and embryonic and extraembryonic potentials. Finally, we discuss pioneering experiments with hPSC-based 3D models that lay the groundwork for understanding how human beings are constructed.

## 2. Key Events in Human Embryogenesis

To comprehend how hPSC-based 3D models can be used to study the processes of morphogenesis beyond implantation in humans, we first need to identify critical points for lineage specification between the blastocyst and pre-gastrula stages, the period when pluripotency rules the embryo.

After the human zygote genome activation at the 4–8 cell stage, in the subsequent 16 cells division (approximately at three days post-fertilization, dpf) begins the specification of the first two embryo lineages: the trophectoderm and inner cell mass (ICM).

Each lineage can be distinguished by the expression and presence of specific transcription factors (TF). For example, Cdx2 (caudal-type homeobox 2) and Gata3 (GATA binding protein 3) are trophectoderm markers, while Oct4 (octamer-binding transcription factor 4) is a marker for the ICM. It was found that apico-basal polarization processes indicate the fate of human blastomeres towards the trophectoderm or ICM. The increase in F-actin following the accumulation of PARP messenger RNA in the apical zone defines polarized cells in human embryos. At the beginning of the morulae stage, all blastomeres express GATA3, but once the compaction and polarization end (4 dpf), only the cells with an apical domain will maintain the expression of this transcription factor and become the precursor cells of the trophectoderm.

In contrast, apolar cells will be the precursor cells of ICM after they have been separated from the apical domain and lose GATA3 expression [8]. Then, a blastocele-like cavity emerges throughout the compaction and polarization process, indicating the beginning of the blastocyst stage (5 dpf). At 6–7 dpf, the blastocyst is composed of three lineages: the trophoblast, the epiblast and the hypoblast (also called primitive endoderm); the latter two are derived from the ICM [9].

The specific NANOG (from ancient Gaelic Tir na nÓg, the land of youth in Irish mythology) TF can identify the epiblast, whereas GATA4 and GATA6 can identify the hypoblast (GATA-binding protein 4 or 6). Interestingly, the segregation of these lineages from the ICM is regulated by the FGF/ERK2 (fibroblast growth factor and extracellular signal-regulated kinase 2) signaling pathway in mice [10]. However, in humans, its inhibition does not change the number of cells that constitute the epiblast or hypoblast, suggesting that the FGF/ERK2 pathway is expendable for the specification of these lineages [11,12].

Single-cell RNA sequencing technology (scRNA-seq) can now elucidate the possible molecular mechanisms regulating lineage specification. Meistermann et al. performed a pseudo-time scRNA-seq analysis of human morulas and blastocysts (3–7 dpf) and predicted the cells’ trajectory to the blastocyst’s three lineages [13]. They proposed that there is an interaction between the epiblast and trophectoderm for the maturation of the latter. In mice, blastocyst adhesion within the implantation site is mediated by the mature mural trophectoderm (located opposite to the epiblast) [14], while in the human, the trophoblast region adjacent to the epiblast (called polar) attaches to the endometrium. Therefore, it is unknown whether the interaction between the polar trophectoderm and epiblast is indispensable for successful implantation, which would be an event specific to humans and other primates.

Blastocyst formation defines the onset of pluripotency, a property possessed only by the epiblast as opposed to the trophectoderm and hypoblast. Pluripotency is traditionally defined as the ability of a cell to differentiate into all the cell types that comprise the three embryonic layers (endoderm, mesoderm and ectoderm), along with the germline [15]. However, this potential does not encompass the formation of extraembryonic tissues such as the placenta and chorion. Nonetheless, this restriction may only apply to mice because human pluripotent stem cells can differentiate into extraembryonic lineages under specific conditions (refer to the following section). Meanwhile, once the mouse blastocyst implants around 5 dpf, pluripotency lasts about a day before gastrulation begins (6.5–9.5).

In contrast, in humans, it remains for a more extended post-implantation period (9–14 dpf), before gastrulation begins. Remarkably, other lineages, including the amniotic epithelium and extraembryonic mesoderm, develop from the primate epiblast during implantation (7.5–8.5 days post fertilization), which is not observed in the mouse model [4]. These lineages could convey signals to the epiblast or other lineages to prepare and initiate morphogenesis during gastrulation.

**Figure 1:**
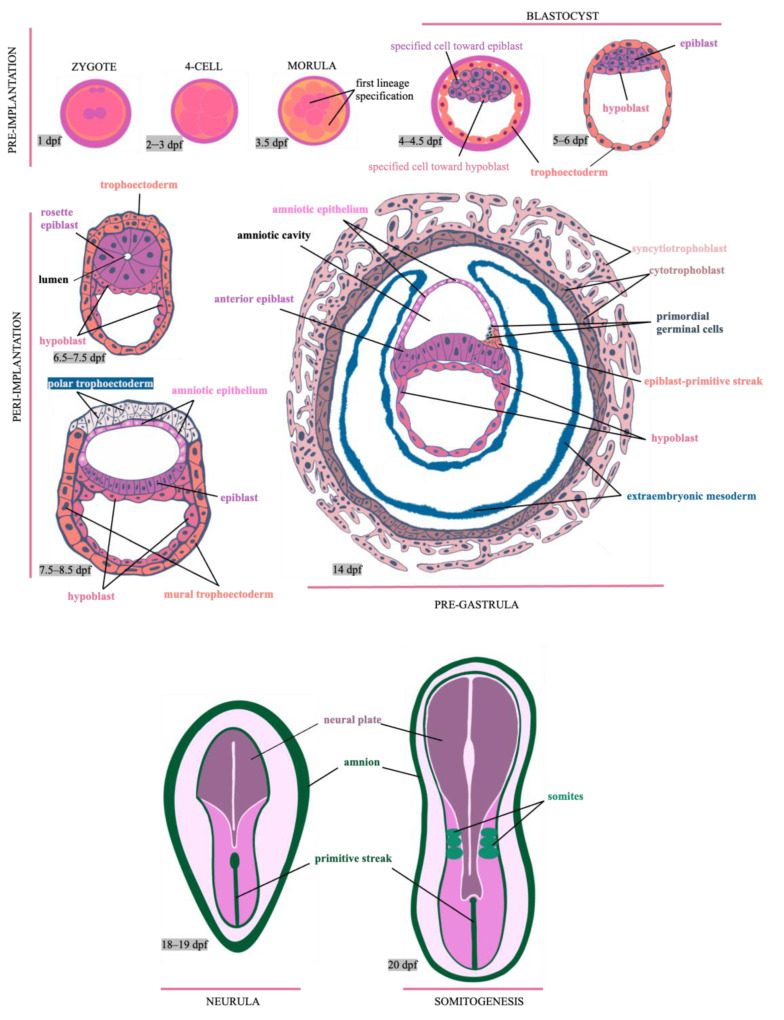
Critical events in early human development. The days post-fertilization (dpf) are an approximation of each embryonic event. One dpf: single cell-zygote stage, with polar bodies. 2–3 dpf: activation of the zygotic genome. 3.5 dpf: morula compaction and first lineage specification (toward trophectoderm and inner cell mass). 4.0–4.5 dpf: early blastocyst containing the outer cells (trophectoderm) (salmon color) and inner cell mass, from which the epiblast (prickly purple color) and hypoblast (shadow azalea pink color) begin to segregate (second lineage specification). 5.0–6.0 dpf: the epiblast (prickly purple color) and hypoblast (shadow azalea pink color) lineages are established. 6.5–7.5 dpf: the start of amniogenesis, where the epiblast (prickly purple color) is arranged in a rosette to form a lumen from which the amniotic cavity arises. The amniotic epithelium derives from a subpopulation of the epiblast. 7.5–8.5 dpf: maturation of the polar trophectoderm (mont blanc peak color), which is essential for the embryo to implant. Probable signals emitted by the epiblast (prickly purple color) or amniotic epithelium (bubblegum kisses color) are required for the maturation of this lineage. 14 dpf: the main embryonic and extraembryonic lineages are established prior to morphogenesis (gastrulation). The trophectoderm differentiated into the outer multinuclear syncytiotrophoblasts (seven veils color) and the mononuclear cytotrophoblasts (natural spring color). The extraembryonic mesoderm (outer reef color) probably arose from the epiblast. The extraembryonic ectoderm from which the chorion is formed is not schematized. The epiblast (prickly purple color) is regionalized (anteroposterior), the formation of the primitive streak starts (orange tea rose color), and the primordial germ cells (aristocratic blue color) are specified, probably from amniotic epithelium (bubblegum kisses color). Dorsal view of an embryo at 18–19 dpf: the primitive streak (kaitoke green color) is already established, indicating the bilateral symmetry of the embryo. The anterior epiblast is differentiated into the neuroectoderm, from which the neural plate (tulipan violet color) arises (named primary neural induction because it is the first induction event in embryogenesis). Finally, the amnion membrane (kaitoke green color) surrounds the embryo. Dorsal view of an embryo at 20 dpf: somites (perky color) begin to emerge (somitogenesis).

Epiblast cells remain pluripotent even after implantation, but their molecular and morphological characteristics readjust themselves in preparation for exit pluripotency and gastrulation. The three embryonic layers and axial patterns are established during this stage. Additionally, the neuroectoderm and the primitive streak (PS) are specified in the epiblast anterior and posterior regions, respectively. In the PS, cells will undergo epithelial–mesenchymal transition (EMT) to form the mesoderm and endoderm lineages [16] (Figure 1).

Nevertheless, developmental processes after implantation remain a “black box” in humans, with minimal information about gastrulation due to the previously mentioned issues. It was until 2021 that an elegant study characterized, for the first time, the human gastrula transcriptome (using an intact embryo around 16–19 dpf, corresponding to Carnegie stage 7), predicting defined lineages such as the epiblast, hypoblast, yolk sac, PS and specific cell populations such as primordial germ cells (PGCs) and hemogenic endothelial progenitors [17]. Although these predictions were based on a single sample with an XY genotype, the study provided valuable insights into pluripotency exit and the diversification of embryonic and extraembryonic lineages during human morphogenesis.

Undeniably, a compendium of developing pathways of human cell lineages, from the zygote to the gastrula, can be obtained by integrating this set with Meistermann’s data [13]. Nonetheless, cellular target maps were constructed using differential gene expression databases from low human embryo samples. Hence, embryonic and extraembryonic lineage trajectories are just hypothetical predictions based on algorithms that must be validated through experimentation.

One possible solution is the hPSC model, specifically using embryonic stem cells (ESCs), as argued in the next section.

## 3. Embryonic Stem Cells and Their Multiple Pluripotent Flavors

Pluripotency during embryonic development is a dynamic phase since the epiblast undergoes different molecular and morphological changes before, during and after implantation without losing its ability to generate the three embryonic layers [18,19]. Nonetheless, pluripotency is fleeting as it vanishes once gastrulation begins. This phase can be captured by ESC line derivation from the adaptation of isolated cells (either from the ICM or epiblast) from blastocysts under specific cell culture conditions.

ESC lines provide valuable insights into the complex development and differentiation mechanisms, allowing us to study how a single embryonic cell can become a fully formed organism. Likewise, with these lines, we were able to inquire about the fleeting epiblast transitions to identify pluripotent stages with specific molecular and potential development profiles, which can differ between species.

The first ESC lines were obtained in 1981 from mouse blastocysts (mESC) [20,21] and fifteen years later from primates (Macaca mulatta and Callithrix jacchus) [22,23]. Years later, researchers derived hESCs from frozen supernumerary embryos, which were procured after consent by suitable informed donors [24]. In 2007, human-induced pluripotent stem cells (hiPSCs) were obtained through reprogramming methods with the pluripotency factors Oct3/4, Sox2 (SRY-box transcription factor 2), Klf4 (Krüppel-like factor 4), and c-Myc [25] (as a note for the reader: the term hPSCs will be used to refer to hESCs and/or hiPSCs).

The adaptations required to maintain the undifferentiated stage of both mESCs and hPSCs are divergent, resulting in data that cannot be extrapolated between species. The first approaches to maintaining mESCs included fetal bovine serum, which promoted heterogeneous cell populations (a state denominated metastable) since it was a temporary period that did not molecularly match the mouse epiblast pluripotency [26]. Therefore, a new experimental set was probed to maintain the mESCs in a molecular state similar to their in vivo counterpart. This new experimental set included a chemically defined medium supplemented with leukemia inhibitor factor (LIF) and the small molecules PD0325901 and CHIR99021, inhibiting ERK2 and GSK3 kinases (mESC LIF/2i condition), respectively. This set represents the “naïve” pluripotency of the pre-implantation epiblast [27,28]. Nevertheless, the epiblast remained pluripotent after implantation but with a different molecular signature (denominated “primed” pluripotency). From it, epiblast stem cells (EpiSCs) were derived [29,30].

The functional difference between the two states can be determined through chimera formation assays, where naïve pluripotent cells can reintegrate into a blastocyst to contribute to the new organism, while primed cells are less competent [31].

Likewise, other discrepancies were reported in the literature, such as differences in the epigenetic chromatin conformation, the imprinted state of the X chromosome, TF expression related to naïve or primed pluripotency [32,33], glycolysis preferentially in primed cells and oxidative phosphorylation for the naive [25,34]. For example, different signaling pathways preserve their undifferentiated state: while naive mESC depend on 2i/LIF; EpiSCs require activation of the FGF and Activin/Nodal pathways [29,35]. Interestingly, hPSCs in “conventional” conditions, such as EpiSCs, do not contribute to chimera formation. They possess a reduced expression of TFs associated with naïve pluripotency, depend on FGF and Activin/Nodal signaling and do not respond to LIF/2i to maintain an undifferentiated state [30]. hPSC transcriptome analysis displays a molecular network like that of the post-implantation human epiblast between 10 and 14 dpf [36], suggesting primed pluripotency. Thus, naïve human pluripotency hunting with different growth factors and small molecules is in progress in the lab (revised in [18,19]).

As previously mentioned, naïve hPSCs show discrepant potential compared to naïve mESCs. The former express trophoblast markers such as KRT7 (keratin 7), TFAP2C (transcription factor AP-2 gamma), TEAD4 (TEA domain transcription factor 4) and GATA3, and differentiate into specialized cells (extravillous trophoblast, syncytiotrophoblast, among others) either through “spontaneous” differentiation or by human trophoblast stem cell derivation [2,37,38].

Naïve hPSCs, through Nodal, WNT, and LIF pathways, produce cells with markers associated with the hypoblast or primitive endoderm (PDGFRA, platelet-derived growth factor receptor alpha; GATA6, GATA binding protein 6; and NID2, nidogen 2) [39]. In contrast, naïve mESCs are not able to differentiate into trophoblast or hypoblast lineages. Thus, the concept of pluripotency in humans should change, referring not only to the derivation of the three embryonic layers but also to a broader potential that includes extraembryonic lineages. This exceptional plasticity of human pluripotency is one of the bases for generating 3D structures similar to the blastocyst and other developmental stages, which will be described in the next section.

## 4. Modeling Human Embryonic Development with Stem Cells—The Building Blocks of Life

Knowledge about pluripotency and in vitro differentiation of hPSCs and mESCs over the last four decades provided an understanding of signaling and transcription mechanisms during development. Nonetheless, these experiments were performed under 2D culture conditions by growing on a feeder layer or synthetic matrices. In the context of embryonic development, cells respond to different types of signals, including interactions between cells or between cells and the extracellular matrix (autocrine or juxtacrine), secreted molecules such as growth factors and morphogens (paracrine) and mechanical forces (mechanotransduction). These signals help to establish the different lineages and the organization of the embryo along various axes. Thus, the complexity observed in vivo embryos is absent in vitro 2D models.

Nevertheless, the differentiation mechanisms are intrinsic to pluripotent lines, and we only need to design the circumstances to direct them toward a more robust organization similar to their in vivo counterparts. Indeed, when both hPSCs and mESCs are seeded in low adherence conditions and without signals to maintain pluripotency (withdrawing FGF2 for hPSCs and 2iL for mESCs), they spontaneously differentiate and form aggregates termed embryoid bodies, which have a particular degree of organization as they incorporate differentiated cells from the three embryonic layers [40]. However, owing to their inconsistent tissue architecture and heterogeneous nature, embryoid bodies provide scarce information about human morphogenesis and embryogenesis.

The 3D-directed differentiation protocols established organoids from hPSC structures, which have an organization analogous to the specific tissue or organ they are trying to resemble [41,42]. A remarkable feature of hPSCs is that they self-organize to generate 3D embryo-like structures [43]. Models representing all embryonic structures, including extraembryonic tissue, are referred to as “integrated models,” whereas models focused on a specific event, such as amniogenesis or somitogenesis, are called “non-integrated models” [44]. It is imperative to discern between integrated and non-integrated models because of ethical and legislative considerations (see the final section in this review), as they can recapitulate whole embryo development to form a viable embryo. However, this statement has not been proven in any laboratory.

## 5. Integrated 3D Embryo Models

### 5.1. Human Blastoids

Wu’s group was a pioneer in blastoid generation, whereby hPSCs were maintained in naïve conditions (2i/LIF plus Activin and FGF2) and were then cultivated in naïve and trophoblast media in a 1:1 ratio with epidermal growth factor (EGF), the activating molecule of the WNT pathway (CHIR99021), two inhibitors of the ALK5 receptor (A-83 and SB-431542) and a histone deacetylase inhibitor (valproic acid). Finally, hPSCs were maintained in a hypoblast medium (FGF2, Activin A and CHIR99021) in AggreWell plates and generated blastocyst-like structures containing an ICM either double-positive for SOX17 (SRY-box transcription factor 17) and GATA6 to identify the hypoblast or SOX2 and KLF17 (Krüppel-like factor 17) to recognize the epiblast, surrounded by external trophoblast-like cells (GATA3, CDX2 and TFAP2C positive) and with a conspicuous cavity. Notably, the efficiency of blastoids generation was only ten percent [36].

Liu and collaborators reported another method to mold blastoids (iBlastoids, since their source was hiPSC). Unlike Yu et al., they reprogrammed human fibroblasts with Oct4, Klf4, Sox2 and c-Myc. After 21 days, the cells formed 3D aggregates, which were CDX2 and GATA2 (trophoblast), NANOG and OCT4 (epiblast) and SOX17 and GATA6 (hypoblast) positive. Their efficiency was 11.5% to that reported in a previous study [45].

Merely identifying embryonic and extraembryonic lineages is insufficient to detect only a few markers associated with these lineages. Strong validation is necessary to determine if blastoids have a transcriptome signature consistent with human blastocysts. However, scRNA-seq analysis revealed the presence of intermediate populations not classified as epiblast, trophoblast or hypoblast, suggesting that they could be an artifact of the reprogramming method. Additionally, trophoblast cells from human blastocysts and blastoids share a similar identity with amnion cells, as they share several markers [46]. In summary, the first wave of human blastoids has limitations, and there is a need to improve the model to represent the embryo accurately.

### 5.2. Blastoids for the Study of Implantation

During implantation, the endometrium becomes “receptive” to allow the adhesion of the embryo so that any alteration can provoke implantation failure (when the trophoblast cells are not able to invade the endometrium) or spontaneous abortion (the embryo implants successfully, but the interface between the placenta and endometrium is subsequently disrupted). Because in vivo implantation is practically impossible to study owing to the issues mentioned, blastoids manifested as an alternative to investigating cellular and molecular processes during this critical event. Kagawa and collaborators generated human blastoids to outline an implantation model. hPSCs were cultured with LIF and ERK2 (PD0325901), WNT (XAV-939) and PKC (Gö 6983) pathway inhibitors to maintain a naïve state. Then, the cells were cultivated with LIF and ALK5 receptor (TGF-β/Nodal pathway, A 83-01), Hippo pathway (1-oleoyl-lysophosphatidic acid), ERK pathway (PD0325901) and ROCK kinase (Y-27632) inhibitors to design blastoids with approximately 70% efficiency. Blastoids were deposited on an endometrial cell layer (glandular epithelial cells), which was previously stimulated with estradiol and progesterone to succeed in their adhesion and copycat implantation. Remarkably, the blastoids adhered only to the receptive endometrial layers, whereas experimental conditions that were not hormonally stimulated or treated with levonorgestrel (a synthetic progestin used as a postcoital contraceptive) failed to implant.

Additionally, aggregates containing only the trophoblast lineage (trophospheres) cannot adhere to the endometrial layer nor mature into the polar trophectoderm [47]. Hence, as demonstrated by an elegant and relatively simple set of experiments, elucidating the interaction between the epiblast and trophoblast cells is essential for embryo development studies. In addition, the avant-garde model could be a platform to study exogenous factors and molecules involved in accurate implantation or pathological situations, which will be discussed later.

### 5.3. Human Embryoids as a Post-Implantation Model

In primates, after implantation, the epiblast segregates into the amnion and embryonic disc; the latter displays a uniform morphology and polarizes along an anteroposterior axis (symmetry rupture). We previously mentioned that PS originates in the most posterior part of the epiblast-embryonic disc, corresponding to the onset of gastrulation. Nevertheless, how is symmetry rupture set up in the human epiblast? In mice, the extraembryonic ectoderm and anterior visceral endoderm (AVE) play essential roles in anteroposterior regionalization. However, we do not know whether these signals originate from the trophoblast, amnion, hypoblast, extraembryonic mesoderm, or all of them in humans [9].

Under the premise that naïve and primed cells represent the preimplantation and post-implantation epiblast, respectively, Simunovic and collaborators designed a 3D model with primed hPSCs to emulate a post-implantation embryo at 12 dpf and detected signals involved in symmetry rupture [48]. Primed hPSCs were induced to form: 1) epiblast-like spheroids in 3D hydrogel culture and 2) extraembryonic-like cells (xEM) by induction with BMP4 (bone morphogenetic protein 4) and FGF2. The xEM-derived cell transcriptome exhibited an amalgam of trophoblast (KRT7, GATA3, GATA2), amnion (WNT6 ‘wnt family member 6,’ ISL1 ‘ISL LIM homeobox’ and GABRP ‘gamma-aminobutyric acid type a receptor subunit pi’) and extraembryonic mesoderm (GATA6, VIM ‘vimentin’ and COL6A1 ‘collagen type VI alpha 1 chain’) markers [48]. Both cellular groups were integrated on low-adherence plates (one or two epiblast-like cysts with 300–500 xEM cells) to generate structures denominated “embryoids”. Astonishingly, half of the embryoids were able to adhere, simulating implantation and showed a columnar epithelium (OCT4 and NANOG positive, epiblast) surrounded by squamous epithelium (amnion) and GATA6+ cells (hypoblast) adjacent to the epiblast-like cells. In addition, they found an outer layer of GATA2+/GATA3+ (trophoblast) cells, which invaded to form a syncytium during implantation. After two–four days in culture, the embryoids had an asymmetric expression, with BRACHYURY and NANOG in the posterior and anterior portions, respectively, which suggests symmetry rupture and PS emergence into the BRACHYURY-positive region. The scRNA-seq confirmed the presence of the PS through MIXL1 (mix1 paired-like homeobox), WNT8A (wnt family member 8A), MESP1 (mesoderm posterior BHLH transcription factor 1) and PDGFRA expression in conjunction with a putative subset of lateral mesoderm progenitors (FOXF1 ‘forkhead box F1’, KDR ‘kinase insert domain receptor’) and paraxial mesoderm (CDX1, CDX2).

On the other hand, the presumptive trophoblast also diversified in subpopulations of the placenta, such as cytotrophoblast (EGFR ‘epidermal growth factor receptor’, BCAM ‘basal cell adhesion molecule’, YAP1 ‘yes1 associated transcriptional regulator’), extravillous trophoblast (ITGA2 ‘integrin subunit alpha 2’, TPM1 ‘tropomyosin 1’, NOTCH ‘neurogenic locus notch homolog protein’) and the mature polar trophectoderm (NR2F2 ‘nuclear receptor subfamily 2 group F member 2’) [48]. Finally, the embryoids did not express AVE lineage markers, which suggests that epiblast regionalization/asymmetry in humans does not depend on AVE as it does in mice.

Therefore, this archetype could elucidate specific post-implantation mechanisms in primates (e.g., the signaling hierarchies that promote PS formation and the rules that underlie cell fate specifications during organogenesis).

### 5.4. The First Ex-Utero Synthetic Embryos

Previous integrated 3D embryo models represent the earliest stages of human embryonic development, up to the onset of the gastrula.

The next challenge is recapitulating organism morphogenesis with all-body plans assembled in 3D models. Current studies are primarily focused on using murine stem cells to develop knowledge for building a synthetic human using stem cells in the future.

One of the main problems in obtaining these “synthetic embryos” is the lack of interactions between different cell types in vitro, as they do during embryonic development. For example, the visceral endoderm is critical for the anteroposterior axis generation of the epiblast by inhibiting signals that “posteriorize” the embryo. Indeed, due to their limited potential to generate extraembryonic endoderm, extraembryonic endoderm (XEN) stem cells have a correspondingly limited ability to contribute to forming embryo-like structures. Zernicka’s group derived an embryo-like structure equivalent to the 6.5 mouse stage, with dorsoventral and anteroposterior axes established from trophoblast stem cells (TSC), murine extraembryonic endoderm stem (XEN) and mESCs co-cultures. Alternatively, they switch the XEN line by XEN induced (iXEN) from mESCs with GATA4 overexpression [49]. However, they failed to advance beyond this stage.

On the other hand, Hanna’s group designed a rotary culture system for the ex-utero culture of mouse embryos post-implantation. They reported the development of embryos from the 5.5- to the 11-day stage, when limbs begin to form [50].

Additionally, they obtained the first stem cells-based “synthetic embryos” with their rotational culture and Zernicka’s reprogramming methods to obtain extraembryonic-type cells. They overexpressed Cdx2 and Gata4 in naive ESCs to obtain naïve inducible Cdx2 and Gata4 mESC (iGata4 and iCdx2 mESC, respectively), which were cultured together with non-transduced naive ESCs in the [51].

In turn, the Zernicka lab also cultured stem cells in Hanna’s bioreactor to obtain pseudo embryos. In one study, mESC were cultured with TSC and iXEN (ETiX) [52], while in another report, the two extraembryonic lineages were reprogrammed from mESC to combine the three epiblast-like, trophectoderm-like and extraembryonic endoderm-like lineages (EiTiX) [53]. These studies described how aggregates self-organized to arrange into anteroposterior and dorsoventral axes of a post-gastrula embryo, recapitulating mouse development between 5.5 and 8.5 dpf. The embryo-like assemblies had extraembryonic membrane components (yolk sac and amnion) and an embryonic compartment with structures that simulate organogenesis (the neural tube, invagination foregut, somites and even a beating heart) such as a mouse embryo at day 8.5; hence, they were called synthetic embryos. However, they lack extraembryonic ectodermal lineages, such as ectoplacental cone cells (EPC) and trophoblast giant cells (TGC) [52,53]. Nevertheless, these fascinating experiments carried out with murine stem cells trace a milestone in experimental embryology by observing the development processes in a pseudo-organism that grows ex-utero. The next challenge is to design the conditions for deriving synthetic embryos from hPSCs.

## 6. Non-Integrated 3D Embryonic Models

### 6.1. Amniogenesis 3D Model

In primates, a subpopulation of the epiblast undergoes an epithelialization process to form an epithelium of squamous cells that ultimately gives rise to the amniotic cavity. Meanwhile, mesothelial cells cover the amniotic epithelium in the trophoblast zone to restrict it between the two layers. In contrast, the epiblast remains pluripotent along the hypoblast edge, forming a columnar pseudostratified epithelium known as the embryonic disc [9].

The molecular and cellular mechanisms involved in human amniotic epithelial cell (hAEC) differentiation from the epiblast are unknown. The importance of their genesis is based on several reasons. In regenerative medicine, hAECs are a possible source of stem cells because when they are isolated from full-term fetal membranes, they present pluripotency-associated markers; therefore, they could be considered a reminder of the epiblast [54]. As previously mentioned, the post-implanted epiblast continues to be pluripotent. However, we must consider that it interacts with the nascent amnion during this period. Therefore, as a gateway to epiblast regionalization and morphogenesis, is this interaction the formula that undercovers the exhaustive pluripotency spectrum in primates?

Fu’s group demonstrated that the amnion is derived from pluripotent cells. They cultured hPSCs in a 3D biomaterial system mimicking the implantation niche with a BMP4 gradient to induce amnion inception. Capable of self-organization, the cells induced an asymmetric embryonic cyst with a lumen (termed PASE, post-implantation amniotic-sac embryoid) and a columnar epithelium (the presumptive epiblast), while the contrary pole exposed to BMP4 produced TFAP2A (transcription factor AP-2 alpha)-positive squamous cells, which considered the amniotic epithelium [55,56].

Another early developmental event that is still unclear is PGC determination in primates. In macaque embryos, the first PGC markers (SOX17, TFAP2C and BLIMP1 ‘B lymphocyte-induced maturation protein-1’) are confined adjacent to the dorsal amnion, which additionally expresses morphogens associated with the germinal induction, such as BMP4 and WNT3, suggesting that primate PGCs are specified from the amnion [57,58]. Thus, during implantation, the posterior epiblast would be more proficient in deriving (in addition to the triad of embryonic layers) lineages such as the amnion or PGC. This is known as “germline pluripotency” and is exclusive to primates [59]. Interestingly, in the PASE model, the pole corresponding to the epiblast-like structure acquires posterior regionalization (simulating the PS), and some cells are TFAP2C+/SOX17+, indicating the formation of PGCs [56]. It should be noted that PASE is an alternative model not only for the study of amniogenesis but also for elucidating the signals required for the germline, either derived directly from the posterior epiblast or that the epiblast has a bifurcation of lineages towards the amnion and PGC, or through an epiblast → amnion → PGC trajectory.

In the most recent work by Fu’s lab, they traced the trajectories of lineages that diversify from the epiblast in their PASE model through single-cell RNA analysis. Epiblast-like cells (EPiLC) branched to derive primitive streak-like cells, amniotic-like cells (AMLC) and primordial germinal cell-like cells (PGCLC), whereas exploring germline origins showed a nascent AMLC population that bifurcated into AMLC and PGCLC [60]. These results suggest that in primates, PGCs are not directly derived from the epiblast or primitive streak. Instead, they support the idea of a subpopulation from the epiblast, which is derived from both the amnion and PGCs, and exhibits “germline pluripotency”.

### 6.2. Formation of Human Gastruloids

The process of gastrulation begins at 6.5 and 16 dpf in mice and humans, respectively, a period where the body plan is established (anteroposterior, dorsal-ventral, among others). Differentiation and morphological partnerships lead the tripartite embryonic lineages to model a spatial location within the axial organization of the embryo. In mammals, gastrulation begins with the formation of the PS. The mesoderm and endoderm progenitors migrate through the PS, undergoing an EMT and derive into the mesoderm and endoderm layers. Moreover, the EMT-avoiding epithelial epiblast forms the ectoderm lineages, such as the neural plate and the epidermis [43]. In mice, gastrulation is mastered by the AVE, PS and extraembryonic ectoderm pathway signals; the latter is derived from the trophectoderm.

The extraembryonic ectoderm induces the visceral endoderm through the BMP pathway [61]. Subsequently, the anterior subpopulation (AVE) blocks the anterior epiblast from “posteriorizing” through BMP/Nodal and WNT pathway antagonists [62,63]. Therefore, mesodermal formation of the PS is restricted in the posterior epiblast. As mentioned, symmetry breaking of a human epiblast in vitro was modeled into the *embryoid*, possibly by interaction with the xEM lineages, although this was not demonstrated by any experimental approach [48]. Nevertheless, it is not necessary to integrate the xEM lineages to trigger gastrulation. Indeed, Moris and collaborators generated gastruloids, which are 3D aggregates from hPSCs. These gastruloids displayed symmetry breaking and a mesodermal differentiation pattern along the anteroposterior axis, comparable to that of the mammalian gastrula. They performed a 24 h pretreatment with CHIR99021 (activating the WNT pathway molecule). Subsequently, the cells were cultured under floating conditions to form aggregates. Interestingly, the aggregate-engineered elongated structures reached maximum dimensions at 96 h, with a precise spatial pattern: first, a BRACHYURY+, followed by a SOX17+, and finally, an SOX2+ domain, which suggested the regionalization of the mesoderm, endoderm and ectoderm lineages, respectively [64].

Assuming that the region where BRACHYURY arose was the most posterior part of the gastruloids, different PS markers were identified, such as CDX2 and CYP26A1 (cytochrome P450 family 26 subfamily A member 1). Interestingly, gastruloids also exhibited an anteroposterior pattern characteristic of somitogenesis, with an expression of “posterior” BRACHYURY, CDX2 and LNFG (lunatic fringe), followed by a short domain of MESP1 and MESP2, and an anterior domain with MEOX1 (mesenchyme homeobox) and TCF15 (transcription factor 15) genes. This somitogenesis feature suggests that gastruloids exhibit Carnegie stage 9 embryo morphology, equivalent to 20–24 dpf [64]. However, in the opposite region of the putative PS, no gene expression associated with the anterior neurectoderm development was detected, but the mesodermal and anterior endoderm lineage patterns continued, such as KDR (kinase insert domain receptor), MEIS1 (meis homeobox 1), MEIS2 (meis homeobox 2), PBX1 (PBX homeobox), TWIST1 (Twist family BHLH transcription factor 1), IRX1, IRX2 and IRX3 (iroquois homeobox 1, 2 and 3). Likewise, in the Simunovic embryoid model, no neural induction markers were detected, although symmetry breaking, and PS onset were described [64]. Ergo, the 3D models that mimic the beginning of gastrulation (gastruloids and embryoids) do not integrate anterior regions such as the neural plate.

### 6.3. 3D Neurulation in Humans

Neurulation begins at the end of gastrulation. The dorsal ectoderm is specified as neural ectoderm and specializes in an epithelium-denominated neural plate, emerging around 7.5 and 18–20 dpf in mice and humans, respectively. The lateral margins of the neural plates then fold inward to transform into the neural tube, which gives rise to the entire central nervous system [65]. Curiously, in each of the models reviewed, the expression of genes related to the formation of anterior structures of the neuroectoderm was absent. Krazburn and collaborators reported that an hPSC-derived 3D neural tube copycat recapitulates aspects of Carnegie stage 8, at around 20–24 dpf. In this framework, the cells were cultured in 2D with a TGF-β inhibitor (SB-431542) for neural induction, and then, BMP4 promoted a regionalization pattern of neural and non-neural lineages of the ectoderm. A synthetic extracellular matrix (Matrigel) forces a transition from 2D to 3D, arranging an epithelium around a lumen (neural tube-like), where anterior neurectoderm [NCAD (N-cadherin), PAX6 (paired box-6), OTX2 (orthodenticle homeobox 2), EMX2 (empty spiracles homeobox 2), SIX3 (six homeobox 3)], non-neural ectoderm [ECAD (E-cadherin), GRHL3 (grainyhead like transcription factor 3) and GATA3], epidermal differentiation [ANXA1–3 (annexin 1-3) or KRT8/18/19 (keratin 8/18/19)], neural crest precursors and migratory cells [SOX10 (SRY-box transcription factor 10), FOXD3 (forkhead box 3), SNAIL1/2 (snail family transcriptional repressor 1/2), TWIST1 (twist family BHLH transcription factor 1)], retinal precursor [SOX4 (SRY-box transcription factor 4), PITX2 (paired like homeodomain 2) and FOXE3 (forkhead box E3)] markers belonging to the neural tube were identified [66]. These data demonstrate in vitro neural tube morphogenesis in humans, which is relevant because neural tube defects have severe consequences for development and are among the most common congenital disabilities (one in every 1000 pregnancies) [67].

### 6.4. Human Somitoids

**Figure 2:**
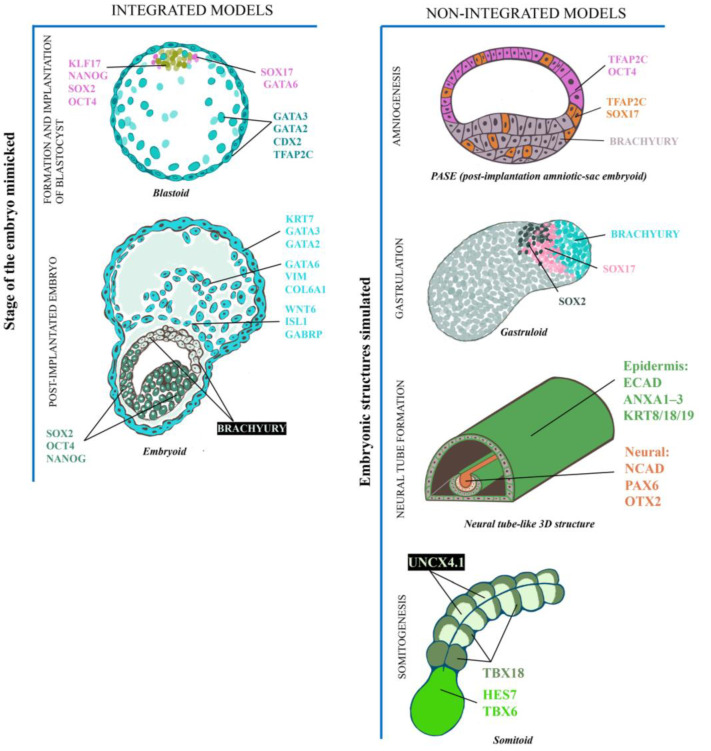
Modeling human embryogenesis with 3D stem cells-based structures. The 3D structures derived from human pluripotent stem cells (embryonic stem cells and induced pluripotent stem cells) are capable of recapitulating both a complete embryo (integrated by embryonic and extraembryonic lineages) as well as events (amniogenesis, gastrulation) or specific structures (neural tube, somites) of the embryo. The blastoids [36,45,47] present the three lineages that make up the blastocyst, each identified by the expression of specific genes (KLF17, NANOG, OCT4 and SOX2 for epiblast-like cells (golden lime color); SOX17 and GATA6 for hypoblast-like cells (purple kush color); GATA3, GATA2, CDX2 and TFAP2C for trophectoderm-like cells (turquoise color). Embryoids [48] presented a columnar epithelium corresponding to epiblast (privet hedge color) (OCT4, NANOG, SOX2) surrounded by a mix of extraembryonic-like cells (pluviophile color) (KRT7, GATA2 and GATA3 for trophectoderm; GATA6, COL6A1 and VIM for extraembryonic mesoderm; ISL1, GABRP, WNT6 for amnion). In this model, the rupture of the symmetry of the epiblast was observed, with the posterior pole positive to BRACHYURY (paradise color), indicating the appearance of the primitive streak. The PASE (post-implantation amniotic sac embryoid) model [55,56] is a cyst with the dorsal side double-positive for OCT4/TFAP2C (amnion-like squamous cells, purple kush color), while the opposite pole is posterior epiblast-like cells (BRACHYURY, resplendent color). Double TFAP2C/SOX17 positive cells indicate the appearance of primordial germ cells (acorn nut color). Gastruloids [64] are elongated structures with specific domains arranged along the anteroposterior axis, indicating the presence of each of the three embryonic layers [BRACHYURY for mesoderm (sea life color), SOX17 for endoderm (begonia pink) and SOX2 for ectoderm (night watch color)]. In the neural tube-like structure [66], the ectoderm cells differentiate into the neural lineages (anterior neuroectoderm, coral gold color), non-neural lineages (epidermis, astroturf color) and neural crest cells (not represented in the scheme). Somitoids [69] are elongated structures of presomitic mesoderm (TBX6, HES7, bitter dandelion color) from which pairs of somite-like segments arise. Each somite-like block exhibited a rostral-caudal organization, demonstrated by the alternating expression of rostral TBX18 (fern canopy color) and caudal UNCX4.1 (frosted garden color) markers.

Somitogenesis also originates during the neurula stage, where transient blocks of the paraxial mesoderm differentiate into the vertebrae, rib cage and skeletal muscle. In humans, somitogenesis is controlled by the segmentation clock, an oscillating molecular mechanism with peaks every 5–6 h [68]. Sanaki-Matsumiya et al. recently reported the creation of organoids containing somite-like structures organized along the anteroposterior axis. hPSCs were treated with a cocktail of FGF2, CHIR99021, SB-431542 and an inhibitor of the BMP pathway (DMH1) to induce the fate of the presomitic mesoderm for 48 h. The cell aggregates were elongated and their differentiation into presomitic mesoderm cells was verified with TBX6 (T-box transcription factor 6), HES7 (Hes Family BHLH transcription factor 7) and LFNG expression, from which pairs of somite-like cell blocks “sprouted”; these were FOXC2 (forkhead box C2), MEOX1 and TFC15 positive. Interestingly, the so-called “somitoid” compartments displayed rostrocaudal and dorsoventral polarity, e.g., sclerotome primordia and dermatomes were also found [69]. The authors highlighted the experimental demand to integrate inductive signals from the whole neurula, such as the neural tube, notochord and mesoderm of the lateral plate (Figure 2).

## 7. 3D Embryonic Models in Clinical Research

According to fetal reprogramming, many pathologies are insulted during early embryonic development and manifest until the postnatal or adult stages. Our understanding of human development can be inferred from the analysis of the embryos of other species. Nevertheless, as mentioned, the exquisite events in primates, such as extraembryonic lineage emergence (amniotic epithelium and extraembryonic mesoderm), do not allow us to extrapolate the knowledge acquired from the classical models (*Danio rerio*, *Xenopus laevis*, *Gallus gallus*, *Mus musculus*). In addition, detailed data of human developmental stages come from a few specimens or even just one (for example, the transcriptome of a single gastrula).

Other milestones, such as human blastocyst adhesion to the uterine epithelium, were inaccessible until the in vitro adhesion experimental setup for blastocysts. However, these experiments cannot be settled routinely; only two research groups worldwide validated this phenomenon. Now, with the synthetic biology of hPSC-derived 3D models, for the first time, we can not only observe otherwise but also manipulate the time window between preimplantation and post-implantation.

Therefore, it is possible to model diseases alluding to implantation and placentation issues related to infertility and early pregnancy loss. The fertility rate is 30 percent and only 50–60 percent continue with gestational development, while 75 percent of interrupted pregnancies are due to implantation failure [70]. A “successful” implantation requires a “tailored dialogue” between the blastocyst and the receptive endometrium between 8 and 9 dpf, orchestrated by numerous molecular components such as hormones, cytokines and growth factors, non-coding RNAs (miRNAs and lncRNAs), epigenetic complexes, immunological factors and extracellular matrix components. At 10 dpf, the blastocyst was embedded in the stroma and mononuclear cytotrophoblasts emerged from the trophoblast layer to initiate placental disposition. An inadequate invasion is associated with pathologies such as preeclampsia and maternal death from hemorrhage. Simultaneously, excessive invasion can lead to excessive placental growth in the uterine wall (*placenta accreta*), extending over the myometrium (*placenta increta*), or even extending beyond the myometrium to cover the uterine serosa and adjacent organs (*placenta percreta*) [70].

On the other hand, according to the fetal reprogramming dogma, we do not comprehend diseases with onset in the pre- and post-implantation periods. Regardless, extensive epidemiological studies suggested that the risk of metabolic disturbances (elevated cholesterol and triglyceride levels, increased body mass index) and neuropsychiatric disorders (increased risk of schizophrenia) in adulthood is associated with exposure to adverse conditions (famine, war, among others) during the first weeks of development [71,72,73,74]. Anencephaly, spina bifida, encephalocele and craniorachischisis are caused by failure of neural tube closure. In the mouse model, mutations in over 200 genes induce neural tube defects. In contrast, a polygenic or oligogenic etiology, along with various non-genetic factors, including histone deacetylase inhibitors (valproic acid or trichostatin A), folic acid antagonists (carbamazepine, fumonisin and trimethoprim) or hyperglycemia (in poorly controlled maternal diabetes mellitus), increase the risk in humans [75,76].

At the same time, the interruption of vertebral segmentation can produce Alagille and Goldenhar syndromes, the Klippel–Feil anomaly, the VACTERL association (V: Vertebral anomalies, A: Anal atresia, C: Cardiovascular anomalies, TE: Tracheoesophageal fistula, R: Renal anomalies, L: Limb defects), spondylocostal dysostosis, congenital scoliosis, kyphosis and lordosis. DLL3 (delta-like canonical Notch ligand 3), PAX1 (paired box 1), SLC35A3 (solute carrier family 35 member A3), WNT3A (Wnt family member 3A), TBX6, LFNG, HES7 and MESP2 mutations were implicated in these malformations. During somitogenesis, the embryo is susceptible to environmental insults such as hypoxia and carbon monoxide exposure, as well as hyperglycemia (gestational diabetes mellitus), hyperthermia, fumonisins, pharmacological blocking agents of potassium channels, phenytoin and valproic acid, agents with teratogenic effects on spinal development [77].

The first approach to evaluate the effects of teratogenic agents during human post-implantation development was reported by Moris et al. (2020). Gastruloids cultured with all-trans retinoic acid (0.4 nM, 33 nM), valproic acid (4 μM, 333 μM), bosentan (4 μM, 18 μM), thalidomide (0.4 μM, 100 μM), phenytoin (20 μM), ibuprofen (63 μM, 250 μM) and penicillin G (63 μM, one mM, two mM). The highest concentrations of retinoic acid, valproic acid and bosentan promoted size reduction, morphological changes and alterations in gene expression (SOX2, neuroectoderm; SOX17, endoderm; and BRACHYURY, mesoderm), while thalidomide and ibuprofen induced alterations no matter the concentration. In contrast, no evident morphological or molecular changes were observed for either penicillin or phenytoin [78].

In summary, hPSC-derived 3D models are used to study the effects and anomalies that occur in gastrulation and to elucidate the multifactorial causes (gene–gene interactions or gene–environmental factors, or teratogenic agents) and possible treatments for several diseases in humans.

## 8. The Ethics Concerning 3D Embryonic Models

We are currently at an unprecedented moment in biology, with new technologies, such as stem cell-based 3D models and high-throughput sequencing platforms, to study embryogenesis and morphogenesis mechanisms in humans. In this way, we are removing the layer of black paint that covers the continuous landscape of human development beyond implantation, which was closed to us for many years and centuries. It was also demonstrated that at the therapeutic potential level, they can be a valuable platform to assess the safety and effectiveness of potential treatments for alterations during development, providing viable solutions. However, scientific progress is often accompanied by various ethical, philosophical, and even legal discussions that concern the scientific community and society in general. In just three years, several reviews were published discussing some of the major ethical dilemmas that arose around 3D models [79,80,81,82,83]. For example, there are ethical considerations when using human 3D embryonic or organoid models in animal experiments for possible transplantation or implantation to test their functionality [80].

Another issue is the informed consent from whom hiPSC lines were derived. Cell donors are usually not advised about generating hiPSC-based-3D embryoid structures and likely commercialization [79,80]. There are also ontological questions about the “nature” of the 3D models; for example, if they were used to recapitulate the development of a human gastrula or neurula as closely as possible, what outcome would ensue? Gastruloids, neuroloids and blastoids can be classified as “artificial embryos”; so, would they have the same moral status that some communities ascribe to embryos? Moreover, recent studies on synthetic mouse embryos demonstrated that cells can self-organize at complexity levels never before observed in vitro. Assuming that the complete ex-utero development of a stem cell-derived embryo will be achieved in the future, will it be considered an autonomous individual, just like the organism that developed in utero under “natural” conditions? Thus, it is necessary to change several concepts of developmental biology, starting with redefining what an embryo is. There is also the question of how much the scientific community has explored and how it should disseminate the findings by studying human embryogenesis using 3D models and emphasizing the potential benefits. If there are inaccurate and incomplete notions, people not versed in this field may have their ethical judgments that lead to concerns and objections (e.g., it is not the same to think of a cortical organoid as a “mini-brain” as “a 3D culture recapitulating developing nerve tissue”).

There is also a discourse on defining ethical and integrity boundaries in research, whereby scientists adhere to established agreements within the scientific community to maintain robust research practices, which pertains to the adequacy of existing regulations and guidelines that govern the utilization of 3D human embryonic models in research in light of recent advances in the field. For example, the culture of gastruloids recalls the stage of 18–21 days pf, which exceeds the period established by the scientific community to study embryos that do not exceed the stage of 14 dpf (although in this scenario, it would be a “like-embryo”). The International Society for Stem Cell Research (ISSCR) guidelines were updated less than two years ago, where non-integrated hPSC-based 3D models (gastruloids, the PASE model, neural tube morphogenesis, somitoids, specific organoids, among others) were classified as Category 1. Projects in this category are exempt from exhaustive revisions, and approval from the Institutional Research and Bioethics Committees is sufficient. In contrast, embryos cultured until 14 dpf or PS formation onset (whichever comes first), as well as 3D models derived from integrated hPSCs, dubbed as those containing embryonic and extraembryonic lineages (blastoids and embryoids), are in Category 2. Thus, research in this category requires approval and is subject to a continuous specialized monitoring process through the coordination of various research, medical and bioethics commissions, including external boards, and must comply with local laws and policies (https://www.isscr.org/isscr-news/the-isscr-releases-updated-guidelines-for-stem-cell-research-and-clinical-translation (accessed on 26 May 2022)).

In response to these questions, 3D models can be deliberately designed to disrupt their potential for developing into a living organism by eliminating or altering individual or multiple components of the equation. In summary, while the potential advantages of utilizing 3D models for studying human embryogenesis are apparent, careful consideration should also be given to the potential risks and concerns.

## 9. Conclusions

Today is a captivating time in embryology, as we are seeing intriguing attempts to unveil the “black box” of human embryogenesis and morphogenesis using hPSC-based 3D models in experiments.

However, several challenges remain, including solving the ethical dilemmas involved in studying these models and designing better culture platforms that allow the integration of different embryonic and extraembryonic lineages. Another challenge is that most analyses performed on 3D embryonic models are only at the DNA or RNA level and bioinformatic analysis. Although several gene clusters were identified as genetic signatures to analyze the spatiotemporal progression of lineages, their functional relevance for the proper development of the embryo has not yet been studied. Contemporary developmental biology provides the valuable potential to other research branches with outstanding human health applications, such as assisted reproduction, tissue bioengineering, and cell therapy in regenerative medicine, fetal programming and translational medicine.

## Data Availability

Not applicable.
